# Explainable artificial intelligence model to predict brain states from fNIRS signals

**DOI:** 10.3389/fnhum.2022.1029784

**Published:** 2023-01-19

**Authors:** Caleb Jones Shibu, Sujesh Sreedharan, KM Arun, Chandrasekharan Kesavadas, Ranganatha Sitaram

**Affiliations:** ^1^Department of Computer Science, University of Arizona, Tucson, AZ, United States; ^2^Division of Artificial Internal Organs, Department of Medical Devices Engineering, Biomedical Technology Wing, Sree Chitra Tirunal Institute for Medical Sciences and Technology, Trivandrum, India; ^3^Department of Imaging Sciences and Interventional Radiology, Sree Chitra Tirunal Institute for Medical Sciences and Technology, Trivandrum, India; ^4^Department of Diagnostic Imaging, St. Jude Children’s Research Hospital, Memphis, TN, United States

**Keywords:** brain state classification, functional near-infrared spectroscopy, brain-computer interface, deep learning, convolutional neural networks, long short-term memory, explainable AI

## Abstract

**Objective:** Most Deep Learning (DL) methods for the classification of functional Near-Infrared Spectroscopy (fNIRS) signals do so without explaining which features contribute to the classification of a task or imagery. An explainable artificial intelligence (xAI) system that can decompose the Deep Learning mode’s output onto the input variables for fNIRS signals is described here.

**Approach:** We propose an xAI-fNIRS system that consists of a classification module and an explanation module. The classification module consists of two separately trained sliding window-based classifiers, namely, (i) 1-D Convolutional Neural Network (CNN); and (ii) Long Short-Term Memory (LSTM). The explanation module uses SHAP (SHapley Additive exPlanations) to explain the CNN model’s output in terms of the model’s input.

**Main results:** We observed that the classification module was able to classify two types of datasets: (a) Motor task (MT), acquired from three subjects; and (b) Motor imagery (MI), acquired from 29 subjects, with an accuracy of over 96% for both CNN and LSTM models. The explanation module was able to identify the channels contributing the most to the classification of MI or MT and therefore identify the channel locations and whether they correspond to oxy- or deoxy-hemoglobin levels in those locations.

**Significance:** The xAI-fNIRS system can distinguish between the brain states related to overt and covert motor imagery from fNIRS signals with high classification accuracy and is able to explain the signal features that discriminate between the brain states of interest.

## Introduction

Brain-Computer Interfaces (BCIs) provide communication pathways between the brain and external devices to be controlled (Wolpaw et al., 2002). BCIs have significant use in motor rehabilitation and communication and can help persons whose motor function or communication is compromised or who cannot communicate with the outside world (Sitaram et al., 2017). Furthermore, providing feedback of brain activity related to specific mental states may help the user control the BCI through operant learning or other forms of learning. The choice of the brain signals acquired and the features selected for feedback form essential steps in BCI development (Benitez-Andonegui et al., 2020; Rieke et al., 2020). BCIs may use electrical and magnetic signals acquired from Electroencephalography (EEG; Stojic and Chau, 2020; Gaur et al., 2021a, b), Magnetoencephalography (MEG; Roy et al., 2020; Ovchinnikova et al., 2021; Rathee et al., 2021), Electrocorticography (ECoG; Luo, 2020; Zhang et al., 2020), or implanted electrodes, or hemodynamic signals acquired from functional Magnetic resonance Imaging (fMRI; Simon et al., 2020; Sorger and Goebel, 2020), or functional near-infrared spectroscopy (fNIRS; Almulla et al., 2020; Li et al., 2020; Nazeer et al., 2020; Shibu et al., 2020; Ghaffar et al., 2021).

fNIRS measures hemodynamic activity by quantifying changes in the concentration of hemoglobin in the brain based on optical intensity measurements. With multiple probes over the subject’s entire scalp, fNIRS can detect hemodynamic activations in the superficial layers of the brain, thus allowing cognitive state determination using techniques such as pattern classification. This neuroimaging modality has recently been utilized to analyze brain functions in both healthy and diseased conditions (Arenth et al., 2007; Irani et al., 2007; Arun et al., 2018). fNIRS is a suitable signal acquisition system for BCI applications owing to its portability, ease of setting up, and robustness to artifacts caused by head motion (Naseer and Hong, 2015).

Coyle et al. (2007) were one of the first to validate the control of a binary switch using mental imagery based fNIRS BCI with an accuracy of above 80% for three subjects (Hong et al., 2015). Sitaram and coworkers could classify the fNIRS signals for motor imagery and motor execution with an accuracy of 80% for five healthy volunteers (Sitaram et al., 2007). Over the last decade, there has been increasing interest in fNIRS as the signal of choice for developing BCI (Naseer and Hong, 2013; Hong et al., 2015; Buccino et al., 2016; Naseer et al., 2016).

One of the broad aims of BCI research is to develop methods that improve the accuracy of brain state classification and ease of implementation. For the past decade, fNIRS-based BCIs primarily focused on extracting informative features from signals and implementing machine learning algorithms like K-Nearest Neighbor (KNN) and Support Vector Machine (SVM). The features included statistical properties like mean (Holper and Wolf, 2011; Faress and Chau, 2013; Naseer and Hong, 2013), signal peak (Bauernfeind et al., 2011; Holper and Wolf, 2011), signal slope (Naseer and Hong, 2013; Hong and Santosa, 2016), signal skewness (Tai and Chau, 2009; Holper and Wolf, 2011), signal kurtosis (Tai and Chau, 2009; Holper and Wolf, 2011), of the fNIRS time domain signals. Filter coefficients and discrete wavelet transforms (DWTs) have also been used (Khoa and Nakagawa, 2008; Abibullaev and An, 2012).

However, the extraction of these statistical features alone may limit classification performance as it depends on the types of features extracted which may not be an exhaustive set of discriminating features. Such manual feature selection is obviated by deep learning techniques which directly process the raw data. Deep learning has gained immense popularity due to its ability to automate feature selection and extraction processes (Chiarelli et al., 2018; Trakoolwilaiwan et al., 2018; Tanveer et al., 2019; Janani et al., 2020).

Deep learning models, along with their ability to extract features and learn from those features, are being used for various applications like classification, detection, and segmentation of images. Convolutional Neural Networks (CNN) are trained commonly for image classification (O’Shea and Nash, 2015; Nguyen et al., 2019; Olmos et al., 2019) and consist of convolutional layers for feature extraction from images. With multiple, fully connected layers, these models can learn to distinguish features of labeled images. Long short-term memory (Hochreiter and Schmidhuber, 1997) is a variant of the recurrent neural network and is designed specifically for time-series data. LSTM has the ability to remember important information for longer periods of time, and hence is most commonly used for sequence prediction, like speech recognition (Ying et al., 2020), human activity recognition (Wang and Liu, 2020), etc.

Trakoolwilaiwan et al. performed a study comparing the classification performance of SVM, Artificial Neural Networks (ANN), and Convolution Neural Networks (CNN). In the above work, the input data to the CNN consisted of changes in HbO- (oxyhemoglobin) and HbR-(deoxyhemoglobin) concentration of all channels available with a dimension of *M* by *N* where *M* is the number of data points and *N* is the number of channels available. Various combinations of 1D CNNs (single and multiple convolutional layers, and varying the number of filter kernels) achieving an average classification of over 89% for discriminating rest, left, and right states (Trakoolwilaiwan et al., 2018).

Nagabushanam et al. (2020) compared Support Vector Machine (SVM) and Long-Short Term Memory (LSTM) classification techniques from EEG signals for various tasks and showed that LSTM performs better than SVM by roughly 15%.

A recent study by Z. Shi et al. (Lu et al., 2020) comparing machine learning models like SVM, KNN, and LDA with deep learning models like long short-term memory-fully convolutional network (LSTM-FCN), found that deep learning models could achieve a decode mental arithmetic task with a classification accuracy of 97%.

In a recent study by Ghonchi et al. (2020), spatiotemporal maps were extracted from fNIRS-EEG signals during a motor imagery task to classify the task conditions by a deep neural network. The authors were able to classify the brain states of motor imagery with an accuracy of 99%.

With the growing ability of complex yet reliable Deep Learning models to classify brain states there seems to be a tradeoff between with accuracy and interpretability of a model’s output. Deep Learning network was in the past a black box, because an explanation in terms of which features affected the trained model to arrive at a specific decision was missing.

The eXplainable Artificial Intelligence (XAI) developed in recent years aims to explain the supervised machine learning models (Gunning and Aha, 2019). A few XAI models in the recent literature are summarized below:

1. Local Interpretable Model-Agnostic Explanations (LIME; Ribeiro et al., 2016) attempts to comprehend the models by varying the input data samples and analyze the changes in predictions. LIME modifies a single input value by changing the feature value and then observes the result. LIME generates a list of explanations that indicate the contribution of each characteristic to the data sample prediction. LIME has a few drawbacks, such as the fact that its explanations are not resilient, meaning that a minor change in the input causes the explanations to alter substantially the importance of features (Alvarez-Melis and Jaakkola, 2018).
2. Deep Learning Important FeaTures (DeepLIFT) DeepLIFT aims to explain the difference between a neural reference and an input (Shrikumar et al., 2017). DeepLIFT is a model explanation that gives input variables a significant score. This score is determined by building a partial derivative-like function that is used in conjunction with the chain rule to track the change in the output layer relative to the input layer.
3. SHAP (SHapley Additive exPlanations) is a method that uses *shapley values* to explain the model’s output (Lundberg and Lee, 2017). *Shapley values* are a concept acquired from game theory where different players in a coalition are analyzed for each player’s contribution to the coalition’s success. DeepSHAP is a fast approximation approach for computing SHAP values in DeepLIFT’s built-in deep learning models. It uses feature significance determined using linear composition rules to explain the output, and the chain rule to back propagate the activation difference from the output layer to the original input.

**Figure 1:**
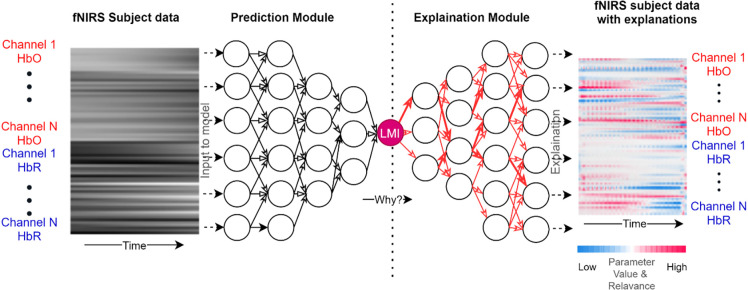
Overview of xAI-fNIRS system. The Deep Learning Model in the prediction module uses the data from each participant in the form of frames as input. The model provides a prediction based on this data, such as 97% LMI (Left Motor Imagery/Left Active). The Deep Learning Predictions are then explained in terms of input variables such as channel 1 HbO, channel 2 HbR, and so on in the Explanation module.

Given its success in recent literature, we propose a SHAP-based explainable AI-fNIRS (xAI-fNIRS) system in our study. We will use this approach: (i) to classify fNIRS signals obtained during overt motor execution tasks as well as covert motor imagery tasks to accurately predict the task; and (ii) to find the contribution of each channel to the model’s performance during classification. The proposed xAI-fNIRS system shown in Figure 1, comprises of a classification Deep Learning model for classifying brain states, during overt motor execution and covert motor imagery conditions which is generalized over two different datasets, followed by an explanation module. The latter consists of DeepSHAP network to introduce explainability to the model output by constructing a decomposition of CNN output on the input variables (i.e., channels).

## Methods

### Data acquisition

#### Dataset A

**Figure 2:**
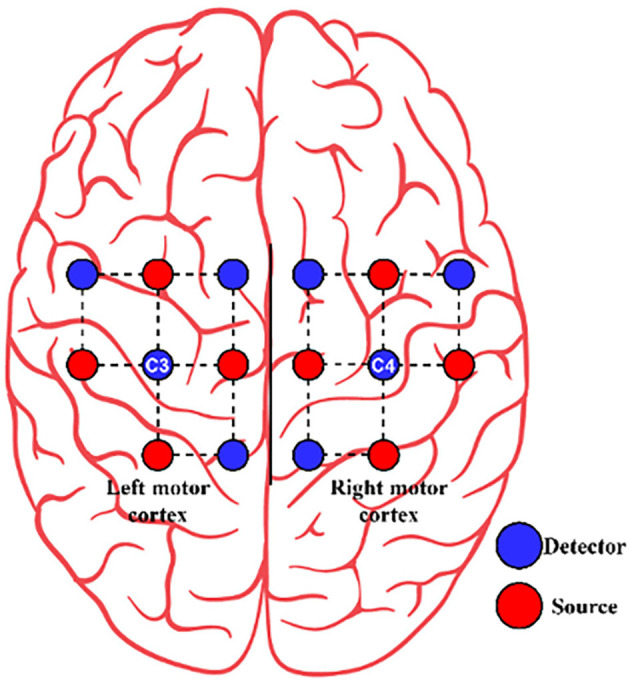
The arrangement of source (red circle) and detector (blue circle) for Dataset A.

Three healthy subjects (Two females and one male) with a mean age of 25 years participated in the study. Ethical clearance was obtained for data acquisition from the Institutional Ethics committee of Sree Chitra Tirunal Institute for Medical Sciences and Technology ethics committee. For data acquisition, a continuous wave, multichannel fNIRS device (NIRsport from NIRx Medical Technologies LLC, Berlin, Germany, RRID: SCR_002491 and NIRStar (v.15.2) software (from NIRx Medical Technologies LLC, Berlin, Germany, RRID: SCR_014540) with two wavelengths (750 nm and 830 nm) and a sampling frequency of 7.8125 Hz, was employed. A total of eight sources and eight detectors were used in combination to form 20 channels. They were placed over bilateral motor cortices for signal acquisition, as shown in Figure 2. The Optode locations are based on the international 10–20 system (Homan et al., 1987). The distance between the source and the detector was 3 cm.

#### Dataset B

We used an open-access dataset from Technische Universität Berlin containing fNIRS motor imagery data. The dataset contained 28 right-handed and one healthy left-handed subjects with average age (years) 28.5 ± 3.7.

There were no neurological, neuropsychiatric, or other brain-related illnesses reported by any of the subjects. The experimental protocol was explained to all volunteers, and informed consent was obtained from all participants (Shin et al., 2017).

**Figure 3:**
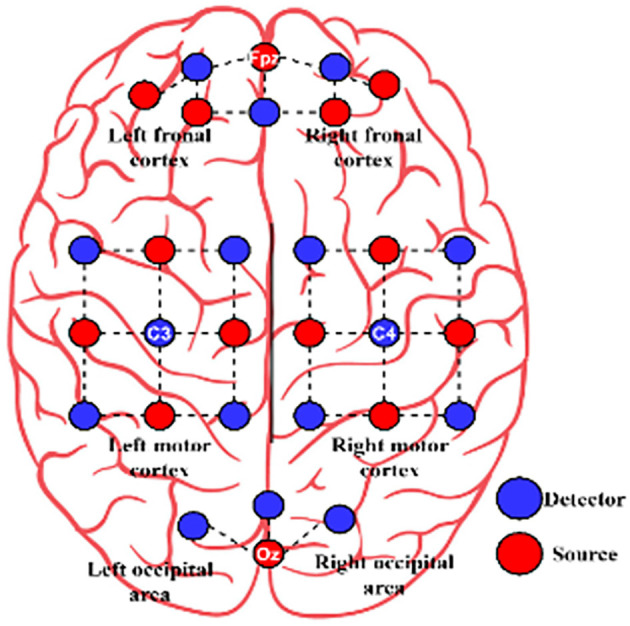
The arrangement of source (red circle) and detector (blue circle) for Dataset B.

At the sampling rate of 10 Hz NIRS data was acquired using NIRScout (NIRx GmbH, Berlin, Germany). One physiological NIRS channel is created by each nearby source-detector pair. At three brain areas, 14 sources and 16 detectors were used to create 36 physiological channels. The frontal (nine channels around Fp1, Fp2, and Fpz), motor (12 channels around C3 and C4, respectively), and visual (three channels around Oz) portions of the brain were studied. (Figure 3).

### Experimental procedure

#### Dataset A

**Figure 4:**
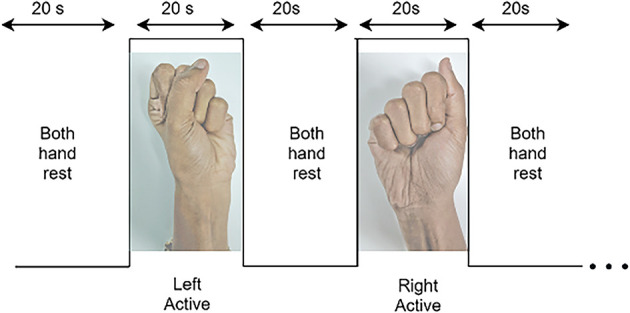
Dataset A: The paradigm for the experimental procedure includes rest and active motor tasks: right- and left-hand motor execution, starting from rest-state on both hands followed by left-hand motor execution task then both hand rest followed by right-hand motor execution.

The subjects were asked to be seated on a chair facing a computer monitor on which the experimental task was displayed. To generate a robust signal of the neural activity, subjects were requested to perform a motor execution. Subjects were instructed to relax before the paradigm began, to stabilize blood flow to the cortex. They were then instructed to relax during the rest period and open and close their left-hand or right-hand palm depending on the screen’s cue. Each subject had four sessions, and each session had 10 rest periods, each following an experimental event. The experiment involved 10 active motor execution tasks (five left and five right), each of 20-s duration separated by 20 s rest periods (Figure 4).

#### Dataset B

**Figure 5:**
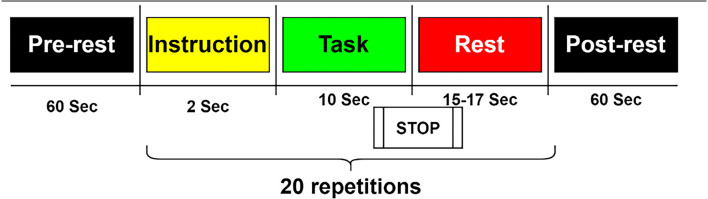
Dataset B: Each session comprised a 1 min pre-experiment resting period, 20 repetitions of the given task, and a 1-min post-experiment resting period. The task started with 2 s of a visual introduction of the task, followed by 10 s of a task period and resting period.

Subjects were asked to engage in kinesthetic motor imagery (imagining opening and closing their hand while grasping a ball). A visual instruction appears on the screen in the form of a black arrow pointing left or right for 2 s. Throughout the activity, MI was performed for 10 s at a time, followed by a 15–17 s rest interval. In a single session, this was done 20 times (10 trials for each left- and right-hand MI in a single session: 30 trials for each one in the whole three sessions). MI tasks were done in a single session based on 10 following blocks, each consisting of one of two conditions: left-hand MI first, then right-hand MI, or* vice versa* (Shin et al., 2017; Figure 5).

### Signal pre-processing

AnalyzIR Toolbox (Santosa et al., 2018) was used to pre-process the fNIRS data. The raw signals were passed through a bandpass filter with a passband of 0.01–0.2 Hz. The filter helps in removing cardiac, respiratory, and other motion artifacts from the signal. Filtered signals were converted to oxygenated (Δc_HbO_(*t*)) and deoxygenated (Δc_HbR_(*t*)) hemoglobin concentration changes using modified Beer-Lamberts Law (Baker et al., 2014):


(1) $$\left[\begin{array}{l} \Delta c_{HbO}\left(t\right)\\ \Delta c_{HbR}\left(t\right) \end{array}\right]=\frac{1}{lxd}{\left[\begin{array}{l} \alpha c_{HbO}\left(\lambda_{1}\right)\;\alpha c_{HbR}\left(\lambda_{1}\right)\\ \alpha c_{HbO}\left(\lambda_{2}\right)\;\alpha c_{HbR}\left(\lambda_{2}\right) \end{array}\right]}^{-1}\left[\begin{array}{l} \Delta A\left(t,\lambda_{1}\right)\\ \Delta A\left(t,\lambda_{2}\right) \end{array}\right]$$

where *ΔA*(*t,λ_j_*), *j* represents unitless absorbance (optical density), which is a measurement of the variation in light emitted at a specific wavelength. *λ_j_*, *αc_HbO_*(λ_1_) and *αc_HbR_*(λ_1_) are the extinction coefficients of HbO and HbR (μ^−1^ mm^−1^) respectively, *d* is the unitless differential path length factor (DPF), and *l* is the distance between the emitter and detector. With the distance between the source and detector by default was maintained at 30 mm, NIRS signal is able to penetrate 15 mm into the gray matter of the brain (Gratton et al., 2006; Zhou et al., 2020; Yücel et al., 2021). Because fNIRS signals are sensitive to motion artifacts like respiration and heartbeat, channels having a coefficient of variation (CV, the standard deviation/mean) of each wavelength exceeding 7.5 were discarded (Minati et al., 2011; Balardin et al., 2017; Pfeifer et al., 2018).

## Background

### Convolutional neural network

**Figure 6:**
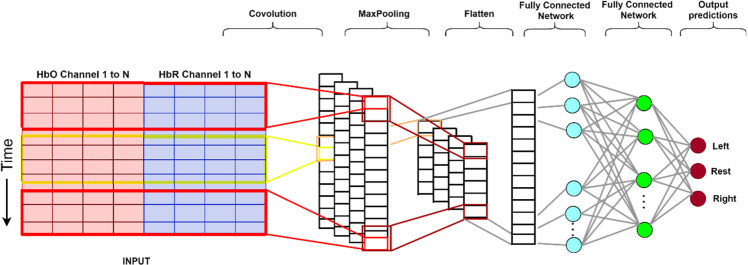
The structure of the CNN used in this study. From the input data (left), the convolutional (encoding) layers extract feature maps. Subsequently, these feature maps are resampled (max-pooling) to a coarser resolution before flattening to a 1-dimensional array. This is then passed on to the first fully connected network which applies weights to predict the correct label. Lastly, the final output layer computes and presents probabilities for each label (Gratton et al., 2006). CNN, convolutional neural network.

The CNN architecture shown in Figure 6 can be divided into four parts (O’Shea and Nash, 2015):

1. The input layer consists of an input matrix, which in our case, on the x-axis represents the fNIRS channels and the y-axis represents the fNIRS time-series data from each channel after windowing.
  The convolutional layer has 32 1D kernels of size 3 (span in time, and spans across all channels), which calculates the neurons’ output for each channel.
  The learnable kernels (filters used to extract features from the image matrix) use a small spatial dimensionality. When the data arrives at the convolutional layer, it convolves each filter across the input’s temporal dimension to produce a 2D feature map. The kernels have a corresponding activation map. Convolutional layers are optimized through three hyper-parameters, namely depth, stride, and zero-padding (Albawi et al., 2017). Depth of an image (assume a color image of dimension 50 × 50 and a depth of 3 which is RGB) or a grayscale video (dimension is height and breadth, and depth are the frames). Each convolutional layer has its own filter of a certain dimension which slides over the input matrix, this is controlled by stride. Stride is the number of pixels shifts over the input matrix. Zero padding is a commonly used modification where symmetrically zeros are added to the input matrix. This is done to preserve the dimension of the input volume in the output volume.
2. The pooling layer down samples along the temporal dimension, reducing the number of parameters within that feature map.
3. The fully connected layers contain neurons directly connected to adjacent layers without connecting to any of the neurons within the same layer. These three layers produce a class score from the activation maps, which are then used for classification.

### Long short-term memory

**Figure 7:**
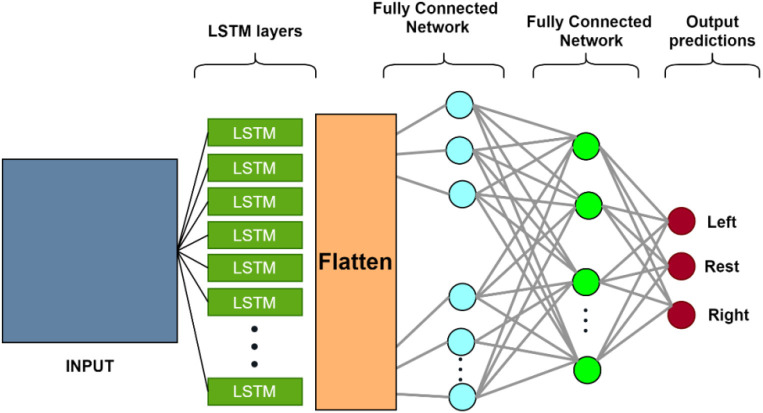
The structure of the LSTM neural network. Each LSTM neuron accepts sequential input data and has three regulators (Input-, output- and forget-gate). It processes the input sequence and determines features based on input data. The remaining structure consists of a dense neural network of three layers (Balardin et al., 2017). LSTM, long short-term memory.

• Long Short-Term memory, shown in Figure 7, is a type of Recurrent Neural Network (RNN) architecture that allows learning from long-term dependencies (Hochreiter and Schmidhuber, 1997; Sutskever et al., 2014; Lipton et al., 2015). The central concept of LSTM is a memory cell that maintains its state over time and is made up of explicit memory (cell state vector) and gating units that control information flow into and out of the memory.
• The LSTM’s memory is represented by the cell state vector, which alters the forgetting of old memory (forget gate) and the addition of new memory (input gate). The output from the previous time step, the current input, and, optionally, the cell status vector are used to govern the flow of information to and from memory.

### Classification metrics

This section explains the strategy used to evaluate the model’s performance using Accuracy, Precision, Recall, and F1 score (Goutte and Gaussier, 2005; Lin et al., 2018; Powers, 2020). First, a brief definition of key terms:

False-positive (FP): the rejection of true null hypothesis; False negative (FN): acceptance of the false null hypothesis; True positive (TP): outcome of the model that correctly predicts observation belonging to the positive class. True negative (TN): outcome of the model that correctly predicts observation belonging to the negative class. Accuracy, precision, recall, and F1 score are computed using these as follows:


(2) $$Accuracy=\left(TP+TN\right)/\left(TP+TN+FP+FN\right)$$

(3) $$\text{Precision}=\text{TP}/\left(\text{TP}+\text{FN}\right)$$

(4) $$\text{Recall = }\text{TP}/\left(\text{TP+FN}\right)$$

(5) $$\text{F1 Score = 2 }\times \left(\text{Precision }\times \text{ Recall}\right)/\left(\text{Precision + Recall}\right)$$

### Proposed sliding window approach

**Figure 8:**
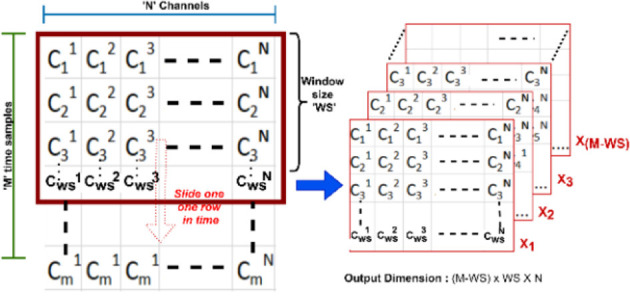
An example of a sliding window approach where M is time in frames, N is the number of channels, and WS is the window size.

A sliding window looks at the latest samples from the signal. fNIRS data is recorded as a time-series with *M* time samples and *N* channels where X_1_ is the latest block of size *WS*, and this block slides one row in time (from row_1_ to row_*M-WS*_) till *X_M-WS_*. This gives an output of dimension [(*M-WS*) × *WS* × *N*] as shown in Figure 8.

**Figure 9:**
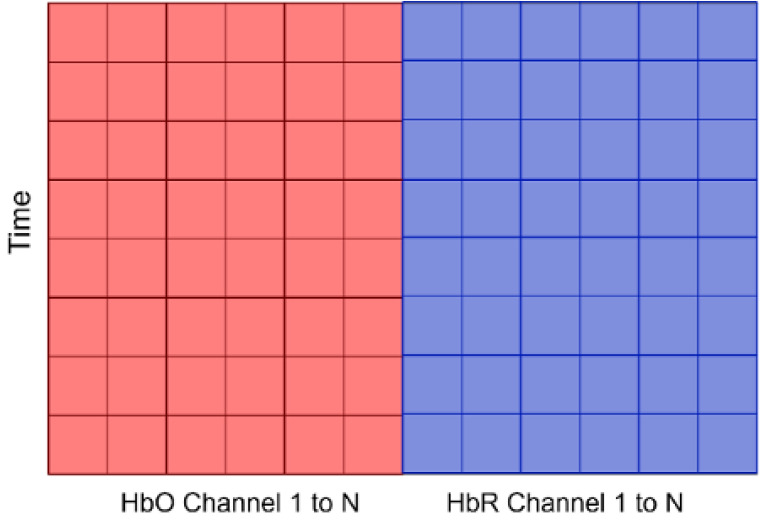
The input data consisting of concentration changes of HbO and HbR in all channels. N for Dataset A varies as we did not have consistent number of channels for all subjects, but for Dataset B, N is 76 (36 for HbO channels and 36 for HbR channels). Time refers to window size.

For our study, we selected a window size of 50, which represents roughly 6 s of data for dataset A and 5 s for dataset B (open access). From the sliding window function’s output, each block’s dimension was computed as 1 × *WS* × *N* which is shown in Figure 9 where time is the *WS* and N is number of channels. Finally, this is labeled as “left,” “rest,” or “right” based on the experimental paradigm.

We followed two approaches to label each block:

Labeling approach 1: Consider a block of specific window size, WS. If, for example, the label “rest” appears in more than 50% of the latest block, then that block is labeled as “rest.” The same method is applied to label blocks as “right” or “left.” For Dataset A this approach has a latency of 4 s, and for Dataset B it has a latency of 5 s.
Labeling approach 2: Consider a block of window size (WS). If all the latest 1 s of labels for this block have a specific label, say left, then that block is labeled as left Likewise, the same is done in case the latest 1 s is labeled as rest or right. For both Dataset A and Dataset B, this approach has a latency of 1 s.

### Proposed structure

For better understanding of the CNN and LSTM models, the input and output sizes of each layer in our proposed CNN model are summarized in Table 1 and likewise for LSTM model are summarized in Table 2. Relu is an activation function that stands for rectified linear unit. If the input is positive, this piecewise linear function will output the input directly; otherwise, it will output a zero (Goodfellow et al., 2016; Arora et al., 2018; Agarap, 2019). Because it is faster to train the model and generally produces high accuracy, most neural networks utilize relu as their default activation function. Softmax is also an activation used in the output layer of the neural network that predicts a multinomial probability distribution for a multi-class classification problem (Goodfellow et al., 2016). Both Dataset A and B used “relu” as the activation function for all layers except the fully connected layer 3 which used a “softmax” as its activation function. Adam is used as an optimization algorithm instead of the classical stochastic gradient descent approach to iteratively update the weights based in the training data (Kingma and Ba, 2017). We used Adam as an optimizer as it has various benefits like easy implementation, computational efficiency, less memory requirements, etc.

**Table 1:** Input and output shapes of the Convolutional Neural Network of this study.

**Layer**	**Input size**	**Output size**	**Properties**
Convolutional Layer 1	50,26	50,32	32 filters with kernel size 3, strides 1
Max Pooling layer 1	50,32	28,32	Pool size 2
Flatten layer 1	28,32	896	
Fully connected layer 1	896	500	500 hidden nodes
Dropout layer 1	500	500	Dropout rate set to 50%
Fully connected layer 2	500	120	120 hidden nodes
Dropout layer 2	120	120	Dropout rate set to 50%
Fully connected layer 3	120	3	3 hidden nodes

**Table 2:** Input and output shapes of Long Short-Term Memory Neural Network of this study.

**Layer**	**Input size**	**Output size**	**Properties**
LSTM layer	50,26	50,120	120 LSTM neurons
Flatten layer 1	50,120	6,000	
Fully connected layer 1	6,000	500	500 hidden nodes
Dropout layer 1	500	500	Dropout rate set to 50%
Fully connected layer 2	500	120	120 hidden nodes
Dropout layer 2	120	120	Dropout rate set to 50%
Fully connected layer 3	120	3	3 hidden nodes

### DeepSHAP explanation module

It is computationally expensive to explore every potential feature combination while working with our datasets. DeepSHAP is a useful tool for estimating Shapley values in deep learning models. We are able to maintain the interpretive capability of Shapley values while maintaining the computational power and accurate outcomes of deep learning models. We can model Shapley values by additive feature attribution techniques for SHAP estimates. Through the explanatory model g, we describe the “coalition vector” *z^′^* for SHAP as follows:


(6) $$g\left(z'\right)=\emptyset_{0}+\sum_{j=1}^{M}\emptyset_{j}Z_{j}^{'}$$

Here, we add the feature attribution for *j*, $\emptyset_{j}$, multiplied by the coalition vector for *j*, $Z_{j}^{'}$, from feature *j* = 1 to the largest coalition size, M. ($z'\in {\left(1,0\right)}^{M}$) is the “coalition vector”. In the coalition vector, each feature is either “present” or “missing” in the combination of characteristics, denoted by a 1 or a 0.

We used DeepSHAP for our explanation module as it is optimized for explaining deep neural networks while having benefits which are:

(a) Global interpretability—The SHAP values can describe how each predictor or input variable contributes, either negatively or positively, to the target variable. This can be used to show each predictor’s positive and negative relationship with the target.
(b) Local interpretability—The SHAP value is assigned to each observation, which increases the transparency by showing how each case is predicted in terms of the contribution of each of the predictors. Traditional interpreters don’t explain individual cases but rather across the entire population. This enables us to pinpoint and contrast the impact of the predictors.

Using SHAP we can also find feature dependence, which is a form of global interpretation plot, where we choose a feature, and plot a point with the feature value on the x-axis and the appropriate Shapley value on the y-axis for each data instance.

Mathematically, we can describe the plot as:${\left(\left(x_{j}^{\left(i\right)},\emptyset_{j}^{\left(i\right)}\right)\right)}_{i=1}^{n}$

With feature dependence we can also find SHAP interaction, which is interaction effects when pairwise features are attributions are considered. It is mathematically given as:


(11) $$\emptyset_{i,j}=\sum_{S\subseteq \left(i,j\right)}\frac{\left|S\right|!\left(M-\left|S\right|-2\right)!}{2\left(M-1\right)!}\delta_{ij}\left(S\right)$$

When *i* ≠ *j*:


(12) $$\delta_{ij}\left(S\right)=\overset{\wedge }{f}\left(S\cup \left(i,j\right)\right)-\overset{\wedge }{f}\left(S\cup \left(i\right)\right)-\overset{\wedge }{f}\left(S\cup \left(j\right)\right)+\overset{\wedge }{f}\left(S\right)$$

Where S represents a subset of all features N except the *i*-th feature and M is the number of input features. This formula calculates the difference between the SHAP values of the *i*-th feature with and without the *j*-th feature and can be used to interpret the SHAP interaction value of the *i*-th feature with respect to the *j*-th feature. This enables us to compute SHAP interaction values using the algorithm for computing SHAP values (Lundberg and Lee, 2017).

## Results

### Hemodynamic response

**Figure 10:**
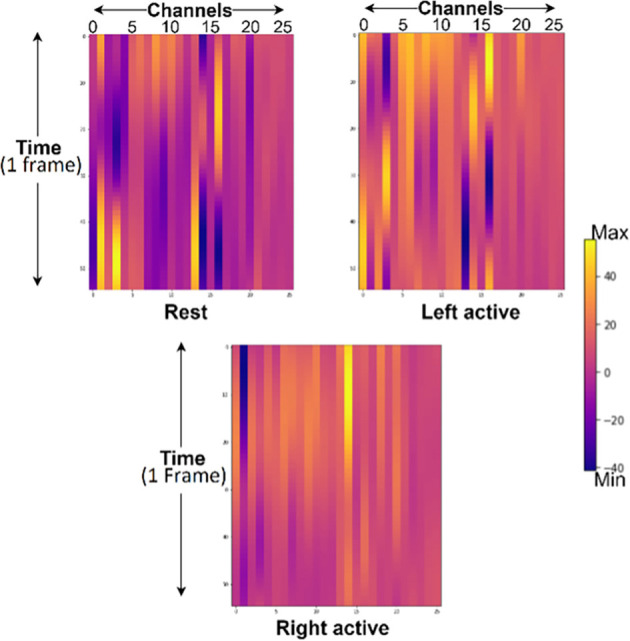
Hemodynamic response from rest, left- and right-motor task blocks of subject 2 from Dataset A where channels 1–13 represents HbO and 14–26 represents HbR. Yellow lines represent maximum, and purple represents minimum values respectively.

The hemodynamic response obtained from subject 2 of Dataset A having 26 channels across all sessions of each task for rest, left, and right motor tasks with a window size of 50 are shown in Figure 10.

Changes in HbO- and HbR-concentration were measured as input to our proposed model.

### Classification accuracies

LSTM and CNN were used to determine classification accuracies for each run of every subject. 10-fold cross-validation was performed on data with training- and validation- data split as 75% for training and 25% for testing.

**Figure 11:**
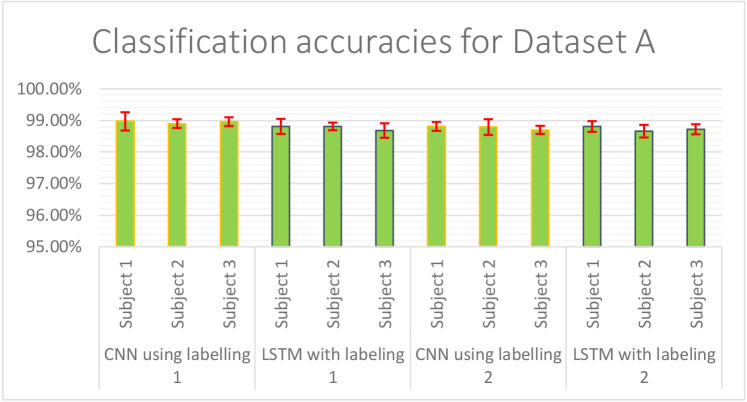
Classification accuracies of CNN and LSTM model using labeling approach -1 and -2 for Dataset A.

**Figure 12:**
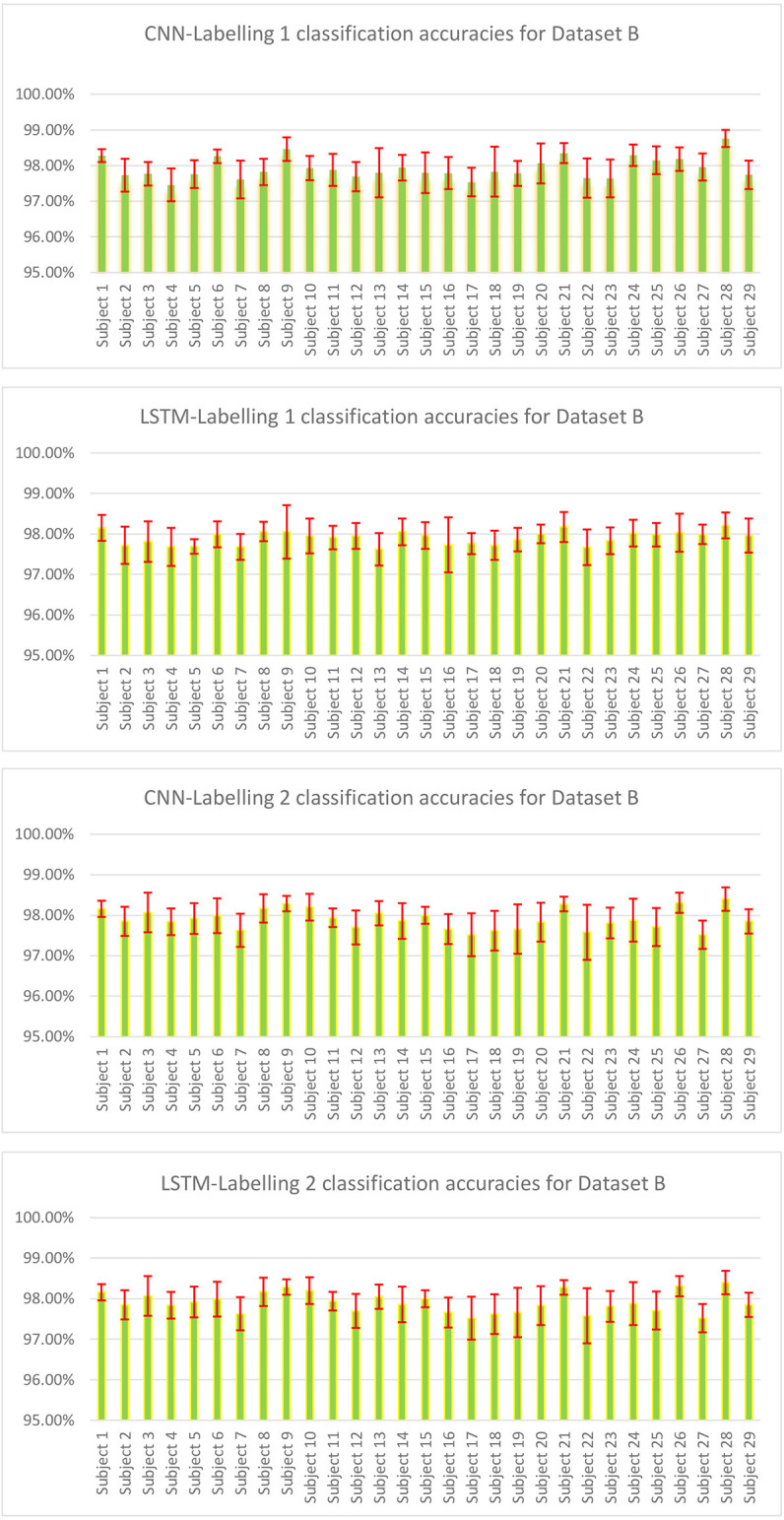
Classification accuracies of CNN and LSTM model for Dataset B where each subject had three sessions and all three were combined for 10-fold cross-validation, and 5-fold cross-validation was done for combined sessions.

The result of validation accuracy for each run of every subject for a window size *WS* of 50 samples (roughly 6 s of data) is shown in Figures 11 and 12 below:

The mean classification accuracy of CNN for Dataset A was 97.68% ± 0.05%, and that for Dataset B was 97.92% ± 0.28%. In LSTM, an average classification accuracy of 98.69% ± 0.04% was recorded for Dataset A and 97.88% ± 0.21% for Dataset B. To test the model for overfitting we randomized the labels. We then performed a permutation test with 10-fold cross-validation on Dataset A with randomized labels, which gave an accuracy of 39.54% ± 1.60% and 36.06% ± 1.88% for CNN and LSTM, respectively. Similarly, 10-fold cross-validated permutation test on Dataset B with randomized labels gave average accuracies of 56.55% ± 0.33% for CNN and 56.62% ± 0.39% for LSTM. Therefore our model is robust.

### Subject independent classification

For subject independent classification we combined a few subjects (a combination of three, five and 10) for training and leaving out one for testing with 10-fold cross validation. The results are shown in Table 3. Dataset A gave an average classification accuracy of 50%. The same couldn’t be done for Dataset B as the number of channels from one subject to another varied.

**Table 3:** Subject independent classification.

**Model**	**Training with subjects**	**Testing with subjects**	**Testing Accuracy**	
			**Labeling Approach 1**	**Labeling Approach 2**
CNN	1 and 2	3	51.06% (+/– 0.09%)	51.62% (+/– 0.11%)
LSTM			49.53% (+/– 0.10%)	47.98% (+/– 0.11%)
CNN	1–3	4	47.99% (+/– 0.10%)	47.98% (+/– 0.08%)
LSTM			48.20% (+/– 0.08%)	44.32% (+/– 0.09%)
CNN	1–4	5	42.30% (+/– 0.11%)	43.30% (+/– 0.07%)
LSTM			46.05% (+/– 0.06%)	42.13% (+/– 0.08%)
CNN	1–5	6	57.22% (+/– 0.08%)	50.13% (+/– 0.12%)
LSTM			53.72% (+/– 0.02%)	48.52% (+/– 0.08%)
CNN	1–6	7	47.33% (+/– 0.10%)	50.55% (+/– 0.08%)
LSTM			50.20% (+/– 0.08%)	46.19% (+/– 0.05%)
CNN	1–7	8	52.93% (+/– 0.06%)	51.10% (+/– 0.11%)
LSTM			53.29% (+/– 0.07%)	52.27% (+/– 0.05%)
CNN	1–8	9	54.87% (+/– 0.08%)	54.12% (+/– 0.09%)
LSTM			56.78% (+/– 0.10%)	53.02% (+/– 0.13%)
CNN	1–9	10	50.10% (+/– 0.09%)	38.38% (+/– 0.07%)
LSTM			48.22% (+/– 0.09%)	42.23% (+/– 0.08%)

### Classification accuracy and validation loss

**Figure 13:**
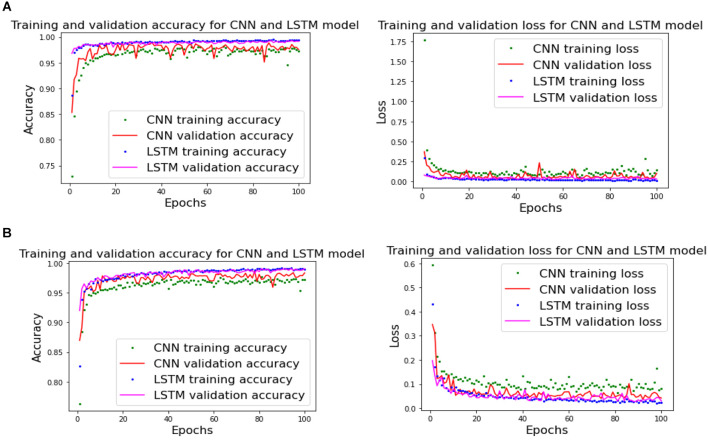
Training- and testing-accuracy and loss for CNN and LSTM. **(A)** Subject 1 of Dataset A and **(B)** Subject 12 of Dataset B.

Figure 13 shows that our model is not overfitting for both CNN and LSTM as training- and validation- accuracies and losses plots converge for each epoch.

### Visualizing the convolutional filters

**Figure 14:**
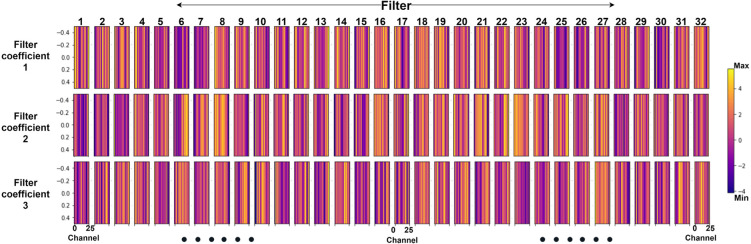
Spatiotemporal features from 32 1-d CNN filters where each filter has three coefficients (Shown for subject 3 with 26 channels for CNN classifier).

Figure 14 shows the CNN model’s features for subject 3 of Dataset A, which is challenging to interpret. SHAP values were used to understand which features contributed the most. This is explained in the next section.

### Explanations

Our studies involved a Deep Learning model, and this necessitated the use of an explainer module. In our study, we used DeepExplainer, a module in DeepSHAP. The DeepExplainer examined the CNN model along with 2,000 random samples of data from the training set. SHAP values were extracted from 1,000 random samples from the test dataset. We obtained a SHAP value of the dimension 3 × 1,000 × 50 × 72 where 3 is the number of classes and 1,000 is the number of random samples of dimension 50 frames of data from 72 channels.

**Figure 15:**
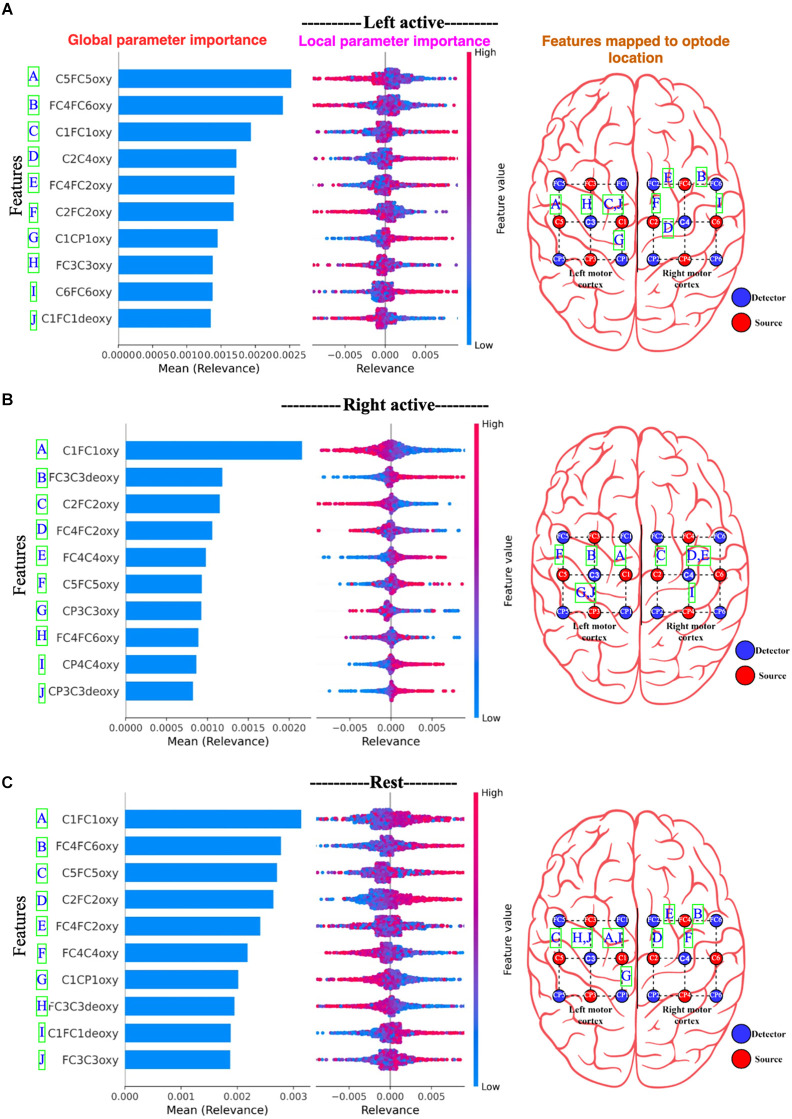
Panel **(A)** represents Left active motor task; **(B)** represents Right active motor task and likewise, **(C)** represents Rest. The global features’ importance and local explanations summary are displayed in the results of the explanation module for Dataset A. Note only 10 channels are plotted. On the right most part, the channels are mapped to the edges of the optode locations.

**Figure 16:**
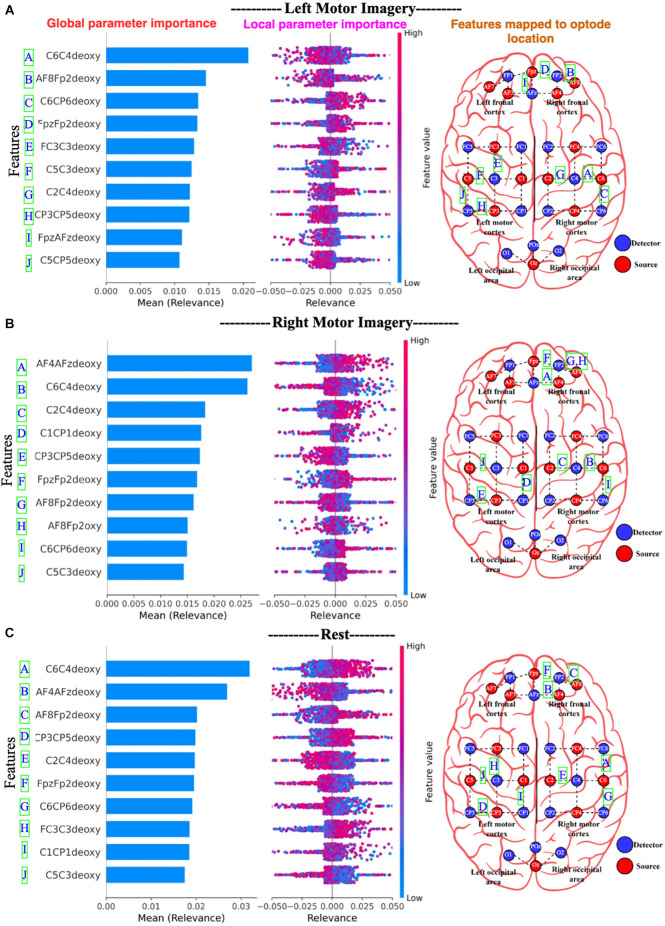
Panel **(A)** represents Left active motor imagery; **(B)** represents Right active motor imagery and likewise, **(C)** represents Rest. The global features’ importance and local explanations summary are displayed in the results of the explanation module for dataset B. Note only 10 channels are plotted. On the right most part, the channels are mapped to the edges of the optode locations.

Figure 15, for Dataset A, the 10 most important channels for each of the three brain states (Left active, right active, and Rest) are shown. Figure 16, for Dataset B, the 10 most important channels for each of the three brain states (LMI, RMI, and Rest) are shown. With respect to deThe Channels are ranked by decreasing mean relevance calculated from local, back-propagated relevance ratings over subject 1 for Dataset A and subject 1 for dataset B.

In Figures 15 and 16, the parameters are displayed as a blue horizontal bar in ascending order of global parameter importance, as determined by mean relevance. All of the individual data points are colored by parameter value and sorted by mean importance in the local explanation summary. The number of data-points at the related level of relevance correlates with the height of the data-points for each feature, as determined by back-propagated relevance scores for each feature. The feature value linked with the local explanation is used to color-code this. The influence of a feature on the model’s categorization is indicated by the position of the dot on the x-axis. Multiple dots that land at the same × location pile up to demonstrate density. For example, Figure 15B Right active (Motor task) seems to associate with the high FC3C3deoxy and low C1FC1oxy from the left cortex. Likewise, Figure 16B Right motor imagery seems to be associated with high AF4AFzdeoxy and C2C4deoxy from the right frontal cortex and right motor cortex. The summary distribution allows us to get an idea of what to expect from the fNIRS signal classification model.

**Figure 17:**
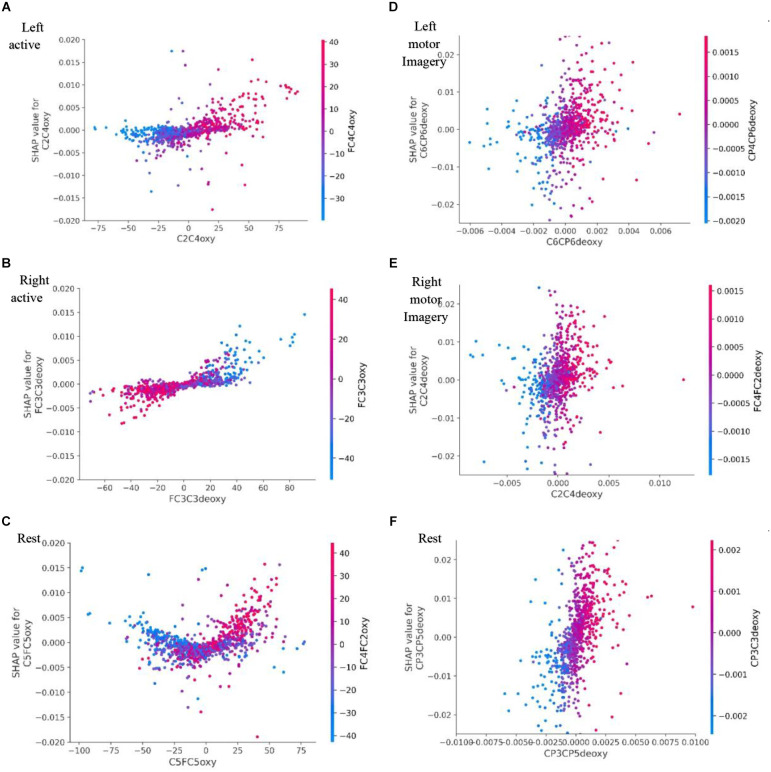
Dataset A’s SHAP dependance plot for: **(A)** represents Left active motor task; **(B)** represents Right active motor task and likewise **(C)** represents Rest. Dataset B’s SHAP dependance plot for: **(D)** represents Left motor imagery; **(E)** represents Right motor imagery and likewise **(F)** represents Rest. Here the SHAP dependence plot where the SHAP value of the feature has a positive effect on the classification is plotted against the value of the feature for all examples over the dataset and it is color coded with the feature it interacts with the most. SHAP, SHapley Additive exPlanations.

Another interesting finding is shown in Figure 17A Left motor imagery and (B) Right motor imagery where high values of channel C6C4deoxy contribute to other classes (rest or motor left imagery) than itself (right motor imagery), but it contributes to Rest state in (C) where it clearly shows the high values for channel C6C4deoxy contribute more towards the classification of Rest state.

The dependence plot shows the marginal effect one or two features have on a Deep Learning model’s predicted outcome. Dependence plots display the relationship between a feature’s value (x-axis) and the prediction of each sample (each dot) in a dataset (y-axis). In comparison to conventional partial dependency charts, they offer deeper information. Figure 17 is a SHAP dependence plot where a feature that has a positive effect on classification is plotted against the value of that feature for all examples in the dataset.

Figure 17B right active, SHAP value of FC3C3deoxy is plotted against its actual value, clear interaction with FC3C3oxy, which increases the classification impact towards right active (Motor task). We can also observe that when the FC3C3deoxy value is low it has a negative impact and classifies anything other than right active, when the FC3C3deoxy value is high it has a positive impact towards classification of right active. Likewise, Figure 17D. Left motor imagery (LMI) channel CP4CP6deoxy shows interaction with channel FC2FC4deoxy, low values CP4CP6deoxy have negative impact towards classification of LMI and high values of CP4CP6deoxy contribute more towards LMI.

## Discussion

Deep Learning models like CNN and LSTM enable automated feature extraction and classification of fNIRS signals. The previous works on fNIRS used statistical features which were extracted for machine learning algorithms (Naseer and Hong, 2013; Naseer et al., 2014), e.g., mean, slope, skewness, etc. of HbO and HbR, which yielded a classification accuracy of 86.19% for SVM whereas CNN enhanced the classification accuracy to 93.08% (Trakoolwilaiwan et al., 2018).

The window-based approach (Naseer and Hong, 2013) restricts attention only to variation in data for contiguous windowed periods, whereas the proposed sliding window approach looks at multiple overlapping windows starting with each input sample.

As data scarcity is one limiting factor of Deep Learning techniques (Ghonchi et al., 2020; Lu et al., 2020; Nagabushanam et al., 2020) the sliding window also augments the data by increasing the data’s sample size, as explained in Section “Proposed sliding window approach”.

The Supplementary Material shows the number of channels available channels in Dataset A, which shows that despite fewer channels, the classification module can classify with an accuracy over 98% accuracy for both CNN and LSTM shown in Figure 12. We used Dataset B, an open-access dataset (Abibullaev and An, 2012) that consisted of motor imagery for 29 subjects to bolster our study. The classification module also gave a classification accuracy of over 98% for both CNN and LSTM for Dataset B, shown in Figure 12.

We trained the model with various window sizes from 1 s to 8 s and 5–6 s (50 frames), which led to the most optimal results. This shows that the longer the sliding window’s duration, the greater the amount of historical data in the window, which contributes to better classification accuracy.

For Dataset A, we could not perform intersubjective group classification as the number of channels did not remain constant for all three subjects. As for Dataset B, we performed intersubjective group classification shown in Supplementary Material; we observe that the accuracy is low due to a large outlier or lack of normalization of optode placement across all subjects.

The spatiotemporal features identified in Figure 14 show the fNIRS signal variation, from which the model has learned features for classification. Each of the filters describes temporal variation for three-time samples and spatial variation along the 26 channels (subject 3 of dataset A); however, it is not interpretable.

To introduce interpretability and trackability in neural networks for BCI purposes so far, few EGG studies have shown that it is possible to represent using direct pixel-to-channel mapping (Joshi et al., 2018), projection of sensor locations by interpolating image of the sensor on the scalp (Bashivan et al., 2015), and an fNIRS study channels with a frequency greater than mean were considered to be most contributing channels another fNIRS study used z-score and t-value methods for channel selection (Nazeer et al., 2020).

SHAP (SHapley Additive exPlanations) is a novel method that employs a game-theoretical approach to describe the output of a Deep Learning model. A recent study used SHAP to explain which channels contributed the most in classifying EEG data for the Active touch task (Alsuradi et al., 2020).

In the explanation module of the “xAI-fNIRS” system we aimed to explain which features contribute to the classification of a specific task in the CNN model for both Dataset A and Dataset Busing DeepSHAP which is a module in SHAP.

From Figures 16 and 17, we observe the high-relevance features (Channels) for Dataset A and Dataset B. We plotted 10 features (channels) that show the highest level of relevance for classification in descending order. An interesting finding is that the explanation module showed that Dataset A had all oxy values of channels that contribute the most to motor task brain states’ classification, whereas Dataset B had all deoxy values of channels for the classification of motor imagery brain states.

It is important to note that the xAI-fNIRS system presented in this study should not be conceived as the one-and-only multi-outcome model. Instead, it should be viewed as a general model for building precise and explainable models towards the development of better BCI systems.

In summary, we have presented the xAI-fNIRS system to classify brain activity states, which can be used for both task and imagery tasks in fNIRS. The xAI-fNIRS shows high classification performance while enabling the possibility of explaining the classification in terms of pinpointing decisive input data to provide BCI researchers to understand the underlying reasons for classification. We hope that our study will lead to developing a better BCI system that could not only perform with high accuracy prediction of brain states but also help in understanding the underlying spatiotemporal activation patterns.

## Conclusion

The xAI-fNIRS system’s classification module showed high classification accuracy of greater than 98% a sliding window approach with Deep Learning models like CNN and LSTM. The sliding window approach keeps a history of signals and, at the same time, increases the dimension of the dataset. The classification module performed well, giving a high classification accuracy of over 98% for two datasets (Motor task and Motor imagery). Deep learning has gotten so good at classifying brain data, but it fails to reveal which features contribute the most to classification. To solve this issue, the explanation module aims to bring insight into reasons for classification by every channel given a color-coded relevance score; a positive relevance score contributes to the true class and vice versa. Hence the xAI-fNIRS system is a step towards building better BCI systems.

## Data Availability Statement

The raw data supporting the conclusion of this article can be made available on request. However, this is subject to the data sharing protocols of SCTIMST, Trivandrum, India.

## Ethics Statement

The studies involving human participants were reviewed and approved by Institutional Ethics Committee (IEC) of the Sree Chitra Tirunal Institute for Medical Sciences and Technology, Trivandrum. The patients/participants provided their written informed consent to participate in this study. Informed consent was obtained from all individual participants included in the study. The clinical trial has been registered with identification no.SCT/IEC/801/AUGUST-2015.

## Author Contributions

CJS developed the xAI part of the Deep Learning network. CJS and SS were involved in the design of the Deep Learning network. All authors were involved in the formulation of the methodology, contributed to the writing of the article and approved the submitted version.

## References

[B1] Abibullaev B. An J. (2012). Classification of frontal cortex haemodynamic responses during cognitive tasks using wavelet transforms and machine learning algorithms. Med. Eng. Phys. 34, 1394–1410. 10.1016/j.medengphy.2012.01.002 22310482

[B2] Agarap A. F. (2019). Deep learning using rectified linear units (ReLU). arXiv [Preprint]. 10.48550/arXiv.1803.08375

[B3] Albawi S. Mohammed T. A. Al-Zawi S. (2017). “Understanding of a convolutional neural network,” in 2017 International Conference on Engineering and Technology (ICET), (Antalya, Turkey), 1–6.

[B4] Almulla L. Al-Naib I. Althobaiti M. (2020). Hemodynamic responses during standing and sitting activities: a study toward fNIRS-BCI. Biomed. Phys. Eng. Express. 6:055005. 10.1088/2057-1976/aba102 33444236

[B5] Alsuradi H. Park W. Eid M. (2020). “Explainable classification of EEG data for an active touch task using shapley values,” in HCI International 2020 - Late Breaking Papers: Multimodality and Intelligence 6, eds Stephanidis C. Kurosu M. Degen H. Reinerman-Jones L. (Cham: Springer International Publishing), 406–416.

[B6] Alvarez-Melis D. Jaakkola T. S. (2018). On the robustness of interpretability methods. arXiv [Preprint]. 10.48550/arXiv.1806.08049

[B7] Arenth P. M. Ricker J. H. Schultheis M. T. (2007). Applications of functional near-infrared spectroscopy (fNIRS) to neurorehabilitation of cognitive disabilities. Clin. Neuropsychol. 21, 38–57. 10.1080/13854040600878785 17366277

[B8] Arora R. Basu A. Mianjy P. Mukherjee A. (2018). Understanding deep neural networks with rectified linear units. arXiv [Preprint]. 10.48550/arXiv.1611.01491

[B9] Arun K. M. Smitha K. A. Rajesh P. G. Kesavadas C. (2018). Functional near-infrared spectroscopy is in moderate accordance with functional MRI in determining lateralisation of frontal language areas. Neuroradiol. J. 31, 133–141. 10.1177/1971400917739083 29072554 PMC5882061

[B10] Baker W. B. Parthasarathy A. B. Busch D. R. Mesquita R. C. Greenberg J. H. Yodh A. G. (2014). Modified Beer-Lambert law for blood flow. Biomed. Opt. Express 5, 4053–4075. 10.1364/BOE.5.004053 25426330 PMC4242038

[B11] Balardin J. B. Zimeo Morais G. A. Furucho R. A. Trambaiolli L. Vanzella P. Biazoli C.. (2017). Imaging brain function with functional near-infrared spectroscopy in unconstrained environments. Front. Hum. Neurosci. 11:258. 10.3389/fnhum.2017.00258 28567011 PMC5434677

[B13] Bashivan P. Rish I. Yeasin M. Codella N. (2015). Learning representations from EEG with deep recurrent-convolutional neural networks. arXiv [Preprint]. 10.48550/arXiv.1511.06448

[B14] Bauernfeind G. Scherer R. Pfurtscheller G. Neuper C. (2011). Single-trial classification of antagonistic oxyhemoglobin responses during mental arithmetic. Med. Biol. Eng. Comput. 49, 979–984. 10.1007/s11517-011-0792-5 21701852

[B15] Benitez-Andonegui A. Burden R. Benning R. Möckel R. Lührs M. Sorger B. (2020). An augmented-reality fNIRS-based brain-computer interface: a proof-of-concept study. Front. Neurosci. 14:346. 10.3389/fnins.2020.00346 32410938 PMC7199634

[B16] Buccino A. P. Keles H. O. Omurtag A. (2016). Hybrid EEG-fNIRS asynchronous brain-computer interface for multiple motor tasks. PLoS One 11:e0146610. 10.1371/journal.pone.0146610 26730580 PMC4701662

[B17] Chiarelli A. M. Croce P. Merla A. Zappasodi F. (2018). Deep learning for hybrid EEG-fNIRS brain-computer interface: application to motor imagery classification. J. Neural Eng. 15:036028. 10.1088/1741-2552/aaaf82 29446352

[B207] Coyle S. M. Ward T. E. Markham C. M. (2007). Brain–computer interface using a simplified functional near-infrared spectroscopy system. J. Neural Eng. 4, 219–226. 10.1088/1741-2560/4/3/007 17873424

[B21] Faress A. Chau T. (2013). Towards a multimodal brain-computer interface: combining fNIRS and fTCD measurements to enable higher classification accuracy. Neuroimage 77, 186–194. 10.1016/j.neuroimage.2013.03.028 23541802

[B22] Gaur P. Gupta H. Chowdhury A. McCreadie K. Pachori R. B. Wang H. (2021a). A sliding window common spatial pattern for enhancing motor imagery classification in EEG-BCI. IEEE Trans. Instrum. Meas. 70, 1–9. 10.1109/TIM.2021.305199633776080

[B23] Gaur P. McCreadie K. Pachori R. B. Wang H. Prasad G. (2021b). An automatic subject specific channel selection method for enhancing motor imagery classification in EEG-BCI using correlation. Biomed. Signal Process. Control 68:102574. 10.1016/j.bspc.2021.102574

[B24] Ghaffar M. S. B. A. Khan U. S. Iqbal J. Rashid N. Hamza A. Qureshi W. S.. (2021). Improving classification performance of four class FNIRS-BCI using Mel Frequency Cepstral Coefficients (MFCC). Infrared Phys. Technol. 112:103589. 10.1016/j.infrared.2020.103589

[B25] Ghonchi H. Fateh M. Abolghasemi V. Ferdowsi S. Rezvani M. (2020). “Spatio-temporal deep learning for EEG-fNIRS brain computer interface,” in 2020 42nd Annual International Conference of the IEEE Engineering in Medicine Biology Society (EMBC), (Montreal, QC, Canada), 124–127. 10.1109/EMBC44109.2020.9176183 33017946

[B26] Goodfellow I. Bengio Y. Courville A. (2016). Deep Learning. Cambridge, MA: MIT Press.

[B27] Goutte C. Gaussier E. (2005). “A Probabilistic interpretation of precision, recall and F-score, with implication for evaluation,” in Advances in Information Retrieval, eds Losada D. E. Fernández-Luna J. M. (Berlin, Heidelberg: Springer), 345–359.

[B28] Gratton G. Brumback C. R. Gordon B. A. Pearson M. A. Low K. A. Fabiani M. (2006). Effects of measurement method, wavelength and source-detector distance on the fast optical signal. Neuroimage 32, 1576–1590. 10.1016/j.neuroimage.2006.05.030 16872842

[B29] Gunning D. Aha D. (2019). DARPA’s explainable artificial intelligence (XAI) program. AI Mag. 40, 44–58. 10.1609/aimag.v40i2.2850

[B30] Hochreiter S. Schmidhuber J. (1997). Long short-term memory. Neural Comput. 9, 1735–1780. 10.1162/neco.1997.9.8.1735 9377276

[B31] Holper L. Wolf M. (2011). Single-trial classification of motor imagery differing in task complexity: a functional near-infrared spectroscopy study. J. Neuroeng. Rehabil. 8:34. 10.1186/1743-0003-8-34 21682906 PMC3133548

[B32] Homan R. W. Herman J. Purdy P. (1987). Cerebral location of international 10-20 system electrode placement. Electroencephalogr. Clin. Neurophysiol. 66, 376–382. 10.1016/0013-4694(87)90206-9 2435517

[B33] Hong K. S. Naseer N. Kim Y. H. (2015). Classification of prefrontal and motor cortex signals for three-class fNIRS-BCI. Neurosci. Lett. 587, 87–92. 10.1016/j.neulet.2014.12.029 25529197

[B34] Hong K. S. Santosa H. (2016). Decoding four different sound-categories in the auditory cortex using functional near-infrared spectroscopy. Hear. Res. 333, 157–166. 10.1016/j.heares.2016.01.009 26828741

[B35] Irani F. Platek S. M. Bunce S. Ruocco A. C. Chute D. (2007). Functional near infrared spectroscopy (fNIRS): an emerging neuroimaging technology with important applications for the study of brain disorders. Clin. Neuropsychol. 21, 9–37. 10.1080/13854040600910018 17366276

[B36] Janani A. Sasikala M. Chhabra H. Shajil N. Venkatasubramanian G. (2020). Investigation of deep convolutional neural network for classification of motor imagery fNIRS signals for BCI applications. Biomed. Signal Process. Control 62:102133. 10.1016/j.bspc.2020.102133

[B19] Joshi R. Goel P. Sur M. Murthy H. A. (2018). “Single trial P300 classification using convolutional LSTM and deep learning ensembles method,” in Intelligent Human Computer Interaction, ed Tiwary U. (Cham: Springer), 3–15.

[B37] Khoa T. Q. D. Nakagawa M. (2008). Functional near infrared spectroscope for cognition brain tasks by wavelets analysis and neural networks. Int. J. Psychol. Behav. Sci. 2, 89–94. 10.5281/zenodo.1082245

[B38] Kingma D. P. Ba J. (2017). Adam: a method for stochastic optimization. arXiv [Preprint]. 10.48550/arXiv.1412.6980

[B39] Li C. Su M. Xu J. Jin H. Sun L. (2020). A between-subject fNIRS-BCI study on detecting self-regulated intention during walking. IEEE Trans. Neural Syst. Rehabil. Eng. 28, 531–540. 10.1109/TNSRE.2020.2965628 31940543

[B40] Lin W. Tong T. Gao Q. Guo D. Du X. Yang Y.. (2018). Convolutional neural networks-based MRI image analysis for the Alzheimer’s disease prediction from mild cognitive impairment. Front. Neurosci. 12:777. 10.3389/fnins.2018.00777 30455622 PMC6231297

[B41] Lipton Z. C. Berkowitz J. Elkan C. (2015). A critical review of recurrent neural networks for sequence learning. arXiv [Preprint]. 10.48550/arXiv.1506.00019

[B42] Lu J. Yan H. Chang C. Wang N. (2020). “Comparison of machine learning and deep learning approaches for decoding brain computer interface: an fNIRS study,” in Intelligent Information Processing X., eds Shi Z. Vadera S. Chang E. (Cham: Springer International Publishing), 192–201.

[B43] Lundberg S. M. Lee S. I. (2017). A unified approach to interpreting model predictions. Adv. Neural Inform. Process. Syst. 30, 4765–4774. 10.48550/arXiv.1705.07874

[B44] Luo S. (2020). RTCOG: online ECoG-based brain-computer interface system for the decoding, synthesis and classification of speech neural signals. Available online at: http://jhir.library.jhu.edu/handle/1774.2/62726.

[B45] Minati L. Visani E. Dowell N. G. Medford N. Critchley H. D. (2011). Variability comparison of simultaneous brain near-infrared spectroscopy (NIRS) and functional MRI (fMRI) during visual stimulation. J. Med. Eng. Technol. 35, 370–376. 10.3109/03091902.2011.595533 21780948 PMC3182558

[B46] Nagabushanam P. Thomas George S. Radha S. (2020). EEG signal classification using LSTM and improved neural network algorithms. Soft Comput. 24, 9981–10003. 10.1007/s00500-019-04515-0

[B47] Naseer N. Hong K. S. (2013). Classification of functional near-infrared spectroscopy signals corresponding to the right- and left-wrist motor imagery for development of a brain-computer interface. Neurosci. Lett. 553, 84–89. 10.1016/j.neulet.2013.08.021 23973334

[B48] Naseer N. Hong K. S. (2015). fNIRS-based brain-computer interfaces: a review. Front. Hum. Neurosci. 9:3. 10.3389/fnhum.2015.00003 25674060 PMC4309034

[B49] Naseer N. Hong M. J. Hong K. S. (2014). Online binary decision decoding using functional near-infrared spectroscopy for the development of brain-computer interface. Exp. Brain Res. 232, 555–564. 10.1007/s00221-013-3764-1 24258529

[B50] Naseer N. Noori F. M. Qureshi N. K. Hong K. S. (2016). Determining optimal feature-combination for LDA classification of functional near-infrared spectroscopy signals in brain-computer interface application. Front. Hum. Neurosci. 10:237. 10.3389/fnhum.2016.00237 27252637 PMC4879140

[B51] Nazeer H. Naseer N. Khan R. A. Noori F. M. Qureshi N. K. Khan U. S.. (2020). Enhancing classification accuracy of fNIRS-BCI using features acquired from vector-based phase analysis. J. Neural Eng. 17:056025. 10.1088/1741-2552/abb417 33055382

[B52] Nguyen A. Kim J. Oh H. Kim H. Lin W. Lee S. (2019). Deep visual saliency on stereoscopic images. IEEE Trans. Image Process. 28, 1939–1953. 10.1109/TIP.2018.2879408 30403631

[B53] O’Shea K. Nash R. (2015). An introduction to convolutional neural networks. arXiv [Preprint]. 10.48550/arXiv.1511.08458

[B54] Olmos R. Tabik S. Lamas A. Pérez-Hernández F. Herrera F. (2019). A binocular image fusion approach for minimizing false positives in handgun detection with deep learning. Info. Fusion 49, 271–280. 10.1016/j.inffus.2018.11.015

[B55] Ovchinnikova A. O. Vasilyev A. N. Zubarev I. P. Kozyrskiy B. L. Shishkin S. L. (2021). MEG-based detection of voluntary eye fixations used to control a computer. Front. Neurosci. 15:619591. 10.3389/fnins.2021.619591 33613182 PMC7892913

[B56] Pfeifer M. D. Scholkmann F. Labruyère R. (2018). Signal processing in functional near-infrared spectroscopy (fNIRS): methodological differences lead to different statistical results. Front. Hum. Neurosci. 11:641. 10.3389/fnhum.2017.00641 29358912 PMC5766679

[B57] Powers D. M. (2020). Evaluation: from precision, recall and F-measure to ROC, informedness, markedness and correlation. arXiv [Preprint]. 10.48550/arXiv.2010.16061

[B58] Rathee D. Raza H. Roy S. Prasad G. (2021). A magnetoencephalography dataset for motor and cognitive imagery-based brain-computer interface. Sci. Data 8:120. 10.1038/s41597-021-00899-7 33927204 PMC8085139

[B18] Ribeiro M. T. Singh S. Guestrin C. (2016). “Why should i trust you?,” in Proceedings of the 22nd ACM SIGKDD International Conference on Knowledge Discovery and Data Mining (New York, NY, USA), 1135–1144. 10.1145/2939672.2939778

[B59] Rieke J. D. Matarasso A. K. Yusufali M. M. Ravindran A. Alcantara J. White K. D.. (2020). Development of a combined, sequential real-time fMRI and fNIRS neurofeedback system to enhance motor learning after stroke. J. Neurosci. Methods 341:108719. 10.1016/j.jneumeth.2020.108719 32439425

[B60] Roy S. Youssofzadeh V. McCreadie K. Prasad G. (2020). Mapping BCI task imagery brain responses using MEG beta power desynchrony effects. Available online at: https://pure.ulster.ac.uk/en/publications/mapping-bci-task-imagery-brain-responses-using-meg-beta-power-des.

[B61] Santosa H. Zhai X. Fishburn F. Huppert T. (2018). The NIRS brain analyzIR toolbox. Algorithms 11:73. 10.3390/a11050073 PMC11218834 38957522

[B62] Shibu C. J. Sreedharan S. KM A. Kesavadas C. (2020). “Comparison of classification performance of handpicked, handcrafted and automated-features for fNIRS-BCI system,” in 2020 5th International Conference on Intelligent Informatics and Biomedical Sciences (ICIIBMS), (Okinawa, Japan), 152–157. 10.1109/iciibms50712.2020.933639228113943

[B63] Shin J. von Lühmann A. Blankertz B. Kim D. W. Jeong J. Hwang H. J.. (2017). Open access dataset for EEG+NIRS single-trial classification. IEEE Trans. Neural Syst. Rehabil. Eng. 25, 1735–1745. 10.1109/TNSRE.2016.2628057 27849545

[B65] Shrikumar A. Greenside P. Shcherbina A. Kundaje A. (2017). Not just a black box: learning important features through propagating activation differences. arXiv [Preprint]. 10.48550/arXiv.1605.01713

[B66] Simon J. Fishbein P. Zhu L. Roberts M. Martin I. (2020). “Functional magnetic resonance imaging-based brain computer interfaces,” in Neural Interface Engineering: Linking the Physical World and the Nervous System, ed Guo L. (Cham: Springer International Publishing), 17–47.

[B67] Sitaram R. Ros T. Stoeckel L. Haller S. Scharnowski F. Lewis-Peacock J.. (2017). Closed-loop brain training: the science of neurofeedback. Nat. Rev. Neurosci. 18, 86–100. 10.1038/nrn.2016.164 28003656

[B68] Sitaram R. Zhang H. Guan C. Thulasidas M. Hoshi Y. Ishikawa A.. (2007). Temporal classification of multichannel near-infrared spectroscopy signals of motor imagery for developing a brain-computer interface. Neuroimage 34, 1416–1427. 10.1016/j.neuroimage.2006.11.005 17196832

[B69] Sorger B. Goebel R. (2020). Real-time fMRI for brain-computer interfacing. Handb. Clin. Neurol. 168, 289–302. 10.1016/B978-0-444-63934-9.00021-4 32164860

[B70] Stojic F. Chau T. (2020). Nonspecific visuospatial imagery as a novel mental task for online EEG-based BCI control. Int. J. Neur. Syst. 30:2050026. 10.1142/S0129065720500264 32498642

[B71] Sutskever I. Vinyals O. Le Q. V. (2014). Sequence to sequence learning with neural networks. arXiv [Preprint]. 10.48550/arXiv.1409.3215

[B72] Tai K. Chau T. (2009). Single-trial classification of NIRS signals during emotional induction tasks: towards a corporeal machine interface. J. Neuroeng. Rehabil. 6:39. 10.1186/1743-0003-6-39 19900285 PMC2779792

[B73] Tanveer M. A. Khan M. J. Qureshi M. J. Naseer N. Hong K. (2019). Enhanced drowsiness detection using deep learning: an fNIRS study. IEEE Access 7, 137920–137929. 10.1109/ACCESS.2019.2942838

[B74] Trakoolwilaiwan T. Behboodi B. Lee J. Kim K. Choi J. W. (2018). Convolutional neural network for high-accuracy functional near-infrared spectroscopy in a brain-computer interface: three-class classification of rest, right- and left-hand motor execution. Neurophotonics 5:011008. 10.1117/1.NPh.5.1.011008 28924568 PMC5599227

[B75] Wang L. Liu R. (2020). Human activity recognition based on wearable sensor using hierarchical deep LSTM networks. Circuits Syst. Signal Process. 39, 837–856. 10.1007/s00034-019-01116-y

[B76] Wolpaw J. R. Birbaumer N. McFarland D. J. Pfurtscheller G. Vaughan T. M. (2002). Brain-computer interfaces for communication and control. Clin. Neurophysiol. 113, 767–791. 10.1016/s1388-2457(02)00057-3 12048038

[B77] Yücel M. A. Lühmann A. v. Scholkmann F. Gervain J. Dan I. Ayaz H.. (2021). Best practices for fNIRS publications. Neurophotonics 8:012101. 10.1117/1.NPh.8.1.012101 33442557 PMC7793571

[B78] Ying W. Zhang L. Deng H. (2020). Sichuan dialect speech recognition with deep LSTM network. Front. Comput. Sci. 14, 378–387. 10.1007/s11704-018-8030-z

[B79] Zhang X. Xiong Q. Dai Y. Xu X. Song G. (2020). An ECoG-based binary classification of BCI using optimized extreme learning machine. Complexity 2020:e2913019. 10.1155/2020/2913019

[B80] Zhou X. Sobczak G. McKay C. M. Litovsky R. Y. (2020). Comparing fNIRS signal qualities between approaches with and without short channels. PLoS One 15:e0244186. 10.1371/journal.pone.0244186 33362260 PMC7757903

