# Supplementary material for: Explainable artificial intelligence model to predict brain states from fNIRS signals
Source: Front Hum Neurosci. 2023 Jan 19;16:1029784. doi: 10.3389/fnhum.2022.1029784 (PMC9892761; doi:10.3389/fnhum.2022.1029784)
Supplement: Supplementary file 1 [file Data_Sheet_1.docx]

**Dataset Description:**

Channels available for subjects from dataset A and B.

**Dataset A:**

**Subject 1:** C5FC5, FC3C3, CP3C3, C1FC1, C1CP1, C2FC2, C2C4, FC4FC2, FC4C4, FC4FC6, CP4C4, C6C4, C6FC6

**Subject 2:** FC3C3, CP3C3, C1FC1, C1CP1, C2C4, FC4FC2, FC4C4, FC4FC6, CP4C4, C6C4, C6FC6

**Subject 3:** C5FC5, FC3C3, CP3C3, C1CP1, C2C4, FC4FC2, FC4C4, FC4FC6, CP4C4, C6C4

**Dataset B:**

**Subject 1 to 29:** AF7Fp1, AF3Fp1, AF3AFz, FpzFp1, FpzAFz, FpzFp2, AF4AFz, AF4Fp2, AF8Fp2, OzPOz, OzO1, OzO2, C5CP5, C5FC5, C5C3, FC3FC5,  FC3C3, FC3FC1, CP3CP5, CP3C3, CP3CP1, C1C3, C1FC1, C1CP1, C2FC2, C2CP2, C2C4, FC4FC2, FC4C4, FC4FC6, CP4CP6, CP4CP2, CP4C4, C6CP6, C6C4, C6FC6

FpN = Frontopolar

FN = Frontal

TN = Mid Temporal

CN = Central

PN = Parietal

ON = Occipital

When N is odd it belongs to the left cortex and when it is even it belongs to the right cortex. Also, the midline optodes are Fz, Cz and Pz.

**Classification accuracies for Dataset A using:**

1. **Labelling approach 1:**

| **Subject** | **CNN** | | | | **LSTM** | | | |
| --- | --- | --- | --- | --- | --- | --- | --- | --- |
|  | Accuracy | Precision | recall | F1 score | Accuracy | Precision | recall | F1 score |
| Subject1 | 98.15% (+/- 0.40%) | 87.26% | 99.96% | 93.18% | 98.28% (+/- 0.18%) | 88.88% | 99.95% | 94.09% |
| Subject2 | 97.59% (+/- 0.33%) | 84.25% | 99.92% | 91.42% | 97.85% (+/- 0.30%) | 88.56% | 99.87% | 93.88% |
| Subject3 | 97.72% (+/- 0.43%) | 82.32% | 99.93% | 90.27% | 97.93% (+/- 0.30%) | 88.61% | 99.80% | 93.87% |
| Subject4 | 97.68% (+/- 0.36%) | 83.88% | 99.97% | 91.22% | 97.91% (+/- 0.28%) | 90.03% | 99.82% | 94.67% |
| Subject5 | 97.92% (+/- 0.22%) | 83.76% | 99.97% | 91.15% | 97.86% (+/- 0.35%) | 89.22% | 99.82% | 94.22% |
| Subject6 | 98.07% (+/- 0.17%) | 86.30% | 99.97% | 92.64% | 97.70% (+/- 0.39%) | 87.57% | 99.85% | 93.31% |
| Subject7 | 97.66% (+/- 0.30%) | 82.63% | 99.93% | 90.46% | 97.43% (+/- 0.39%) | 86.65% | 99.75% | 92.74% |
| Subject8 | 98.02% (+/- 0.39%) | 84.97% | 99.99% | 91.87% | 98.09% (+/- 0.17%) | 90.21% | 99.87% | 94.79% |
| Subject9 | 98.14% (+/- 0.21%) | 90.70% | 99.90% | 95.08% | 98.35% (+/- 0.37%) | 92.84% | 99.84% | 96.21% |
| Subject10 | 98.06% (+/- 0.38%) | 88.31% | 99.94% | 93.76% | 98.10% (+/- 0.28%) | 90.55% | 99.87% | 94.98% |
| Subject11 | 97.61% (+/- 0.39%) | 81.78% | 99.96% | 89.96% | 98.02% (+/- 0.29%) | 87.34% | 99.84% | 93.17% |
| Subject12 | 97.97% (+/- 0.34%) | 85.36% | 99.96% | 92.08% | 97.73% (+/- 0.39%) | 86.80% | 99.81% | 92.85% |
| Subject13 | 97.85% (+/- 0.56%) | 85.86% | 99.96% | 92.38% | 97.89% (+/- 0.33%) | 87.65% | 99.88% | 93.37% |
| Subject14 | 97.79% (+/- 0.39%) | 85.26% | 99.94% | 92.02% | 98.03% (+/- 0.40%) | 89.45% | 99.87% | 94.37% |
| Subject15 | 97.90% (+/- 0.41%) | 85.22% | 99.98% | 92.01% | 98.05% (+/- 0.18%) | 89.10% | 99.88% | 94.18% |
| Subject16 | 97.64% (+/- 0.47%) | 82.83% | 99.94% | 90.58% | 97.78% (+/- 0.28%) | 89.00% | 99.71% | 94.05% |
| Subject17 | 97.53% (+/- 0.36%) | 88.97% | 99.49% | 93.94% | 97.78% (+/- 0.38%) | 87.92% | 99.78% | 93.47% |
| Subject18 | 97.69% (+/- 0.38%) | 86.28% | 99.89% | 92.59% | 98.00% (+/- 0.42%) | 88.40% | 99.83% | 93.76% |
| Subject19 | 97.82% (+/- 0.36%) | 85.65% | 99.91% | 92.23% | 97.86% (+/- 0.22%) | 87.71% | 99.84% | 93.38% |
| Subject20 | 98.25% (+/- 0.48%) | 87.84% | 99.92% | 93.49% | 97.76% (+/- 0.43%) | 90.47% | 99.75% | 94.88% |
| Subject21 | 98.24% (+/- 0.44%) | 89.81% | 99.96% | 94.62% | 98.03% (+/- 0.35%) | 89.43% | 99.92% | 94.38% |
| Subject22 | 97.68% (+/- 0.29%) | 84.23% | 99.86% | 91.39% | 97.56% (+/- 0.30%) | 88.68% | 99.61% | 93.83% |
| Subject23 | 97.72% (+/- 0.36%) | 83.30% | 99.99% | 90.88% | 97.85% (+/- 0.25%) | 89.85% | 99.76% | 94.55% |
| Subject24 | 98.19% (+/- 0.36%) | 86.78% | 99.97% | 92.91% | 98.19% (+/- 0.25%) | 89.28% | 99.89% | 94.29% |
| Subject25 | 98.05% (+/- 0.34%) | 85.31% | 99.96% | 92.06% | 98.09% (+/- 0.15%) | 90.00% | 99.85% | 94.67% |
| Subject26 | 98.01% (+/- 0.25%) | 87.43% | 99.95% | 93.27% | 97.96% (+/- 0.40%) | 89.06% | 99.86% | 94.15% |
| Subject27 | 97.86% (+/- 0.31%) | 84.59% | 99.97% | 91.64% | 97.91% (+/- 0.33%) | 89.49% | 99.77% | 94.35% |
| Subject28 | 98.59% (+/- 0.19%) | 91.68% | 99.95% | 95.64% | 98.37% (+/- 0.36%) | 91.37% | 99.93% | 95.46% |
| Subject29 | 97.96% (+/- 0.42%) | 84.69% | 99.97% | 91.70% | 97.74% (+/- 0.40%) | 90.21% | 99.74% | 94.73% |

1. **Labelling approach 2:**

| **Subject** | **CNN** | | | | **LSTM** | | | |
| --- | --- | --- | --- | --- | --- | --- | --- | --- |
|  | **Accuracy** | **Precision** | **recall** | **F1 score** | **Accuracy** | **Precision** | **recall** | **F1 score** |
| Subject1 | 97.85% (+/- 0.36%) | 99.77% | 90.32% | 94.81% | 98.08% (+/- 0.33%) | 99.76% | 92.26% | 95.86% |
| Subject2 | 98.07% (+/- 0.49%) | 99.87% | 90.75% | 95.09% | 97.75% (+/- 0.47%) | 99.78% | 90.44% | 94.88% |
| Subject3 | 97.84% (+/- 0.33%) | 99.73% | 90.37% | 94.82% | 97.84% (+/- 0.18%) | 99.81% | 90.42% | 94.88% |
| Subject4 | 97.92% (+/- 0.38%) | 99.84% | 89.08% | 94.15% | 97.85% (+/- 0.31%) | 99.85% | 90.84% | 95.13% |
| Subject5 | 97.99% (+/- 0.43%) | 99.86% | 90.16% | 94.76% | 97.77% (+/- 0.40%) | 99.78% | 90.54% | 94.94% |
| Subject6 | 97.63% (+/- 0.41%) | 99.72% | 88.45% | 93.75% | 97.85% (+/- 0.34%) | 99.83% | 91.39% | 95.42% |
| Subject7 | 98.17% (+/- 0.35%) | 99.90% | 90.80% | 95.13% | 97.65% (+/- 0.29%) | 99.69% | 90.26% | 94.74% |
| Subject8 | 98.29% (+/- 0.19%) | 99.80% | 93.12% | 96.35% | 98.07% (+/- 0.20%) | 99.87% | 91.29% | 95.39% |
| Subject9 | 98.20% (+/- 0.33%) | 99.82% | 92.10% | 95.80% | 98.33% (+/- 0.47%) | 99.92% | 91.64% | 95.60% |
| Subject10 | 97.94% (+/- 0.23%) | 99.80% | 89.01% | 94.10% | 98.12% (+/- 0.39%) | 99.83% | 92.44% | 95.99% |
| Subject11 | 97.70% (+/- 0.42%) | 99.76% | 89.40% | 94.30% | 97.88% (+/- 0.33%) | 99.83% | 90.51% | 94.94% |
| Subject12 | 98.05% (+/- 0.30%) | 99.84% | 89.85% | 94.59% | 97.68% (+/- 0.68%) | 99.78% | 90.29% | 94.80% |
| Subject13 | 97.86% (+/- 0.44%) | 99.74% | 91.06% | 95.20% | 97.75% (+/- 0.35%) | 99.81% | 90.17% | 94.75% |
| Subject14 | 98.00% (+/- 0.21%) | 99.88% | 90.22% | 94.81% | 97.90% (+/- 0.30%) | 99.85% | 90.65% | 95.03% |
| Subject15 | 97.66% (+/- 0.37%) | 99.81% | 89.18% | 94.20% | 98.16% (+/- 0.49%) | 99.80% | 92.15% | 95.82% |
| Subject16 | 97.52% (+/- 0.53%) | 99.73% | 85.20% | 91.90% | 97.61% (+/- 0.25%) | 99.75% | 89.46% | 94.33% |
| Subject17 | 97.62% (+/- 0.49%) | 99.80% | 89.37% | 94.30% | 97.95% (+/- 0.23%) | 99.84% | 90.58% | 94.99% |
| Subject18 | 97.66% (+/- 0.61%) | 99.78% | 89.79% | 94.53% | 97.80% (+/- 0.28%) | 99.78% | 90.15% | 94.72% |
| Subject19 | 97.83% (+/- 0.48%) | 99.90% | 89.90% | 94.63% | 97.87% (+/- 0.37%) | 99.81% | 89.69% | 94.48% |
| Subject20 | 98.28% (+/- 0.18%) | 99.85% | 92.52% | 96.05% | 97.99% (+/- 0.28%) | 99.83% | 91.87% | 95.69% |
| Subject21 | 97.58% (+/- 0.68%) | 99.86% | 87.69% | 93.38% | 98.03% (+/- 0.43%) | 99.85% | 91.46% | 95.47% |
| Subject22 | 97.81% (+/- 0.38%) | 99.81% | 89.44% | 94.34% | 97.52% (+/- 0.48%) | 99.72% | 89.30% | 94.22% |
| Subject23 | 97.88% (+/- 0.53%) | 99.82% | 90.48% | 94.92% | 97.85% (+/- 0.30%) | 99.87% | 90.70% | 95.06% |
| Subject24 | 97.71% (+/- 0.47%) | 99.80% | 90.57% | 94.96% | 97.90% (+/- 0.36%) | 99.88% | 90.93% | 95.19% |
| Subject25 | 98.31% (+/- 0.25%) | 99.72% | 93.43% | 96.47% | 97.96% (+/- 0.34%) | 99.83% | 91.79% | 95.64% |
| Subject26 | 97.52% (+/- 0.35%) | 99.83% | 88.00% | 93.54% | 97.86% (+/- 0.68%) | 99.69% | 92.43% | 95.92% |
| Subject27 | 98.40% (+/- 0.29%) | 99.86% | 93.15% | 96.39% | 97.56% (+/- 0.36%) | 99.66% | 90.21% | 94.70% |
| Subject28 | 97.85% (+/- 0.30%) | 99.84% | 89.22% | 94.23% | 98.34% (+/- 0.22%) | 99.81% | 93.40% | 96.50% |
| Subject29 | 97.85% (+/- 0.36%) | 99.77% | 90.32% | 94.81% | 97.88% (+/- 0.35%) | 99.81% | 90.99% | 95.20% |

**Classification accuracies for Dataset B:**

1. **Labeling approach 1:**

| **Subject** | **CNN** | | | | **LSTM** | | | |
| --- | --- | --- | --- | --- | --- | --- | --- | --- |
|  | Accuracy | Precision | recall | F1 score | Accuracy | Precision | recall | F1 score |
| Subject1 | 98.97% (+/- 0.29%) | 98.96% | 98.98% | 98.97% | 98.81% (+/- 0.24%) | 98.80% | 98.81% | 98.81% |
| Subject2 | 98.90% (+/- 0.14%) | 98.90% | 98.90% | 98.90% | 98.81% (+/- 0.12%) | 98.81% | 98.81% | 98.81% |
| Subject3 | 98.96% (+/- 0.14%) | 98.95% | 98.97% | 98.96% | 98.68% (+/- 0.23%) | 98.68% | 98.69% | 98.69% |

1. **Labeling approach 2:**

| **Subject** | **CNN** | | | | **LSTM** | | | |
| --- | --- | --- | --- | --- | --- | --- | --- | --- |
|  | Accuracy | Precision | recall | F1 score | Accuracy | Precision | recall | F1 score |
| Subject1 | 98.81% (+/- 0.14%) | 98.81% | 98.83% | 98.82% | 98.81% (+/- 0.17%) | 98.80% | 98.83% | 98.81% |
| Subject2 | 98.79% (+/- 0.25%) | 98.79% | 98.80% | 98.80% | 98.66% (+/- 0.20%) | 98.65% | 98.66% | 98.66% |
| Subject3 | 98.70% (+/- 0.13%) | 98.69% | 98.71% | 98.70% | 98.72% (+/- 0.16%) | 98.72% | 98.73% | 98.73% |

**Subject independent classification:**

1. **Dataset A:**

| Model | Training  with subjects | Testing  with subject | Testing Accuracy | |
| --- | --- | --- | --- | --- |
|  |  |  | Labeling Approach 1 | Labeling Approach 2 |
| CNN | 1 & 2 | 3 | 51.06% (+/- 0.09%) | 51.62% (+/- 0.11%) |
| LSTM |  |  | 49.53% (+/- 0.10%) | 47.98% (+/- 0.11%) |
| CNN | 1 to 3 | 4 | 47.99% (+/- 0.10%) | 47.98% (+/- 0.08%) |
| LSTM |  |  | 48.20% (+/- 0.08%) | 44.32% (+/- 0.09%) |
| CNN | 1 to 4 | 5 | 42.30% (+/- 0.11%) | 43.30% (+/- 0.07%) |
| LSTM |  |  | 46.05% (+/- 0.06%) | 42.13% (+/- 0.08%) |
| CNN | 1 to 5 | 6 | 57.22% (+/- 0.08%) | 50.13% (+/- 0.12%) |
| LSTM |  |  | 53.72% (+/- 0.02%) | 48.52% (+/- 0.08%) |
| CNN | 1 to 6 | 7 | 47.33% (+/- 0.10%) | 50.55% (+/- 0.08%) |
| LSTM |  |  | 50.20% (+/- 0.08%) | 46.19% (+/- 0.05%) |
| CNN | 1 to 7 | 8 | 52.93% (+/- 0.06%) | 51.10% (+/- 0.11%) |
| LSTM |  |  | 53.29% (+/- 0.07%) | 52.27% (+/- 0.05%) |
| CNN | 1 to 8 | 9 | 54.87% (+/- 0.08%) | 54.12% (+/- 0.09%) |
| LSTM |  |  | 56.78% (+/- 0.10%) | 53.02% (+/- 0.13%) |
| CNN | 1 to 9 | 10 | 50.10% (+/- 0.09%) | 38.38% (+/- 0.07%) |
| LSTM |  |  | 48.22% (+/- 0.09%) | 42.23% (+/- 0.08%) |

1. **Dataset B:** We couldn’t perform subject independent classification as the number of channels from one subject to another varied.
